# Acute Systemic Complications Following High‐Dose Semaglutide Reinitiation in an Elderly Patient

**DOI:** 10.1002/ccr3.72356

**Published:** 2026-04-23

**Authors:** Jay Mehta, Brent Tai, Johnathan Frunzi

**Affiliations:** ^1^ Internal Medicine Residency Program BayCare Health/Mease Countryside Hospital Safety Harbor Florida USA

**Keywords:** drug holiday, drug‐related side effects and adverse reactions, elderly, esophagitis, glucagon‐like peptide‐1 receptor agonists, hypertensive urgency, semaglutide

## Abstract

Severe gastrointestinal intolerance with systemic complications can occur within hours of reinitiating high‐dose semaglutide after a prolonged treatment interruption, even in patients who previously tolerated the medication. Elderly patients with comorbidities may be particularly vulnerable. Clinicians should consider re‐titration from lower doses when restarting GLP‐1 receptor agonists after drug holidays to minimize severe adverse effects.

## 1 Background

Glucagon‐like peptide‐1 receptor agonists (GLP‐1 RAs), including semaglutide, are widely used for managing obesity and type 2 diabetes. While mild gastrointestinal adverse effects are common during initial dose escalation, severe complications necessitating hospitalization are rarely reported. Even less commonly described is the acute development of severe systemic complications following the reinitiation of therapy after a prolonged drug holiday. Elderly patients with multiple comorbidities and polypharmacy may be especially vulnerable to such adverse effects.

This case illustrates an unusual adverse effect pattern: acute, multi‐system gastrointestinal intolerance following the abrupt reinitiation of high‐dose semaglutide after a two‐month treatment interruption, leading to esophagitis, metabolic derangements, and hypertensive urgency in an elderly patient. This rare presentation has important implications for general internists and subspecialty physicians managing GLP‐1 RA therapy in complex patient populations.

## 2 Case Report

We present a woman in her 80s with hypertension, atrial fibrillation (managed with apixaban and amiodarone), depression, anxiety, migraines, chronic back pain, bladder incontinence, and obesity who presented to the emergency department in September 2025 with a sudden onset of severe gastrointestinal symptoms.

The patient had been prescribed semaglutide for weight management, initially receiving 1 mg subcutaneously weekly in September 2024, with escalation to 1.7 mg weekly in December 2024, and further escalation to 2.4 mg weekly in March 2025. She subsequently discontinued the medication and remained off therapy for approximately 2 months. The previous evening, she had reinitiated semaglutide at the 2.4 mg dose without re‐titration. Within hours, she experienced persistent nausea, vomiting, and severe diarrhea, followed by an intense throbbing headache and progressive weakness.

Upon arrival, the patient was alert and oriented but appeared distressed. Her blood pressure was markedly elevated (systolic greater than 210 mmHg), and she was mildly tachycardic. Her temperature was normal, and oxygen saturation consistently remained above 95% on room air. Physical examination revealed a soft, non‐distended abdomen with diffuse tenderness but no guarding or rebound tenderness.

**FIGURE 1:**
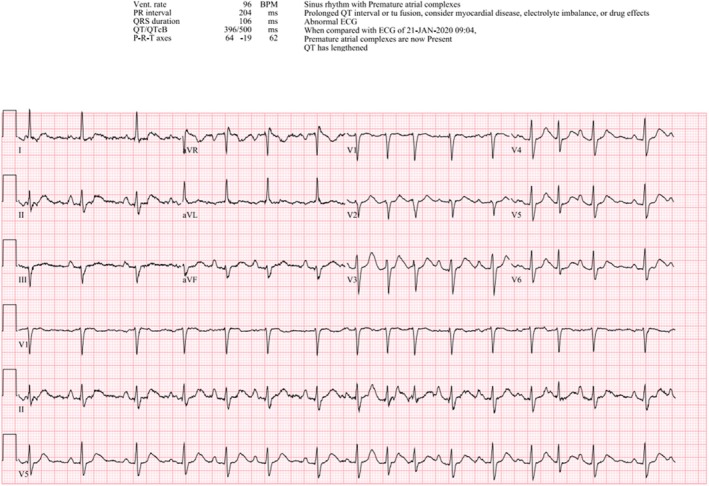
Electrocardiogram showing sinus rhythm with premature atrial complexes and prolonged corrected QT interval (QTc 500 ms) in the setting of severe gastrointestinal losses and hypokalemia. This guided antiemetic selection and electrolyte repletion with close monitoring.

Laboratory investigations revealed neutrophilic leukocytosis (white blood cell count 20.8 × 10^9^/L), hypokalemia (3.0–3.4 mmol/L), and mild thrombocytopenia (135,000 platelets/μl) (Table 1). An electrocardiogram demonstrated a prolonged corrected QT interval (QTc 500 ms), which guided antiemetic selection and prompted aggressive electrolyte repletion with close monitoring (Figure 1). Procalcitonin was low (less than 0.05 ng/mL), and lactate was normal (1.5 mmol/L), making infection unlikely. Chest radiography revealed no acute abnormalities. Urinalysis showed proteinuria (3+) and elevated white blood cells (11–15 per high‐power field), but subsequent urine culture grew only mixed flora consistent with contamination (Table 2).

**TABLE 1:** Complete blood count (CBC) and metabolic panel results on admission. Results demonstrate electrolyte balance, as well as renal and hepatic function parameters, with reference ranges provided for comparison.

Test	Result	Reference range
WBC	20.8 × 10^9^/L (Neutrophil Predominant)	4.0–11.0 × 10^9^/L
Hemoglobin	12.6 g/dL	12.0–15.5 g/dL (for women)
Platelets	135,000 platelets/μL	150,000–450,000 platelets/μL
Potassium	3.0–3.4 mmol/L	3.5–5.0 mmol/L
Chloride	94.9 mmol/L	96–106 mmol/L
Creatinine	0.72 mg/dL	0.59–1.04 mg/dL
eGFR	74–88 mL/min/1.73 m^2^	≥ 60 mL/min/1.73 m^2^
Lactate	1.5 mmol/L	0.5–2.2 mmol/L
Procalcitonin	< 0.05 ng/mL	< 0.05 ng/mL
Capillary glucose	7.3–7.5 mmol/L	3.9–5.5 mmol/L
Lipase	2.1 U/L	0–160 U/L

**TABLE 2:** Urinalysis findings. Initial urinalysis results, including chemical and microbiologic examination parameters, with reference values indicated.

Test	Result	Reference range
Protein	3+	Negative—Trace
WBCs	11–15/HPF	0–5/HPF
Bacteriuria	None	—
Culture (post‐urinalysis)	Mixed flora (contaminant)	—

**FIGURE 2:**
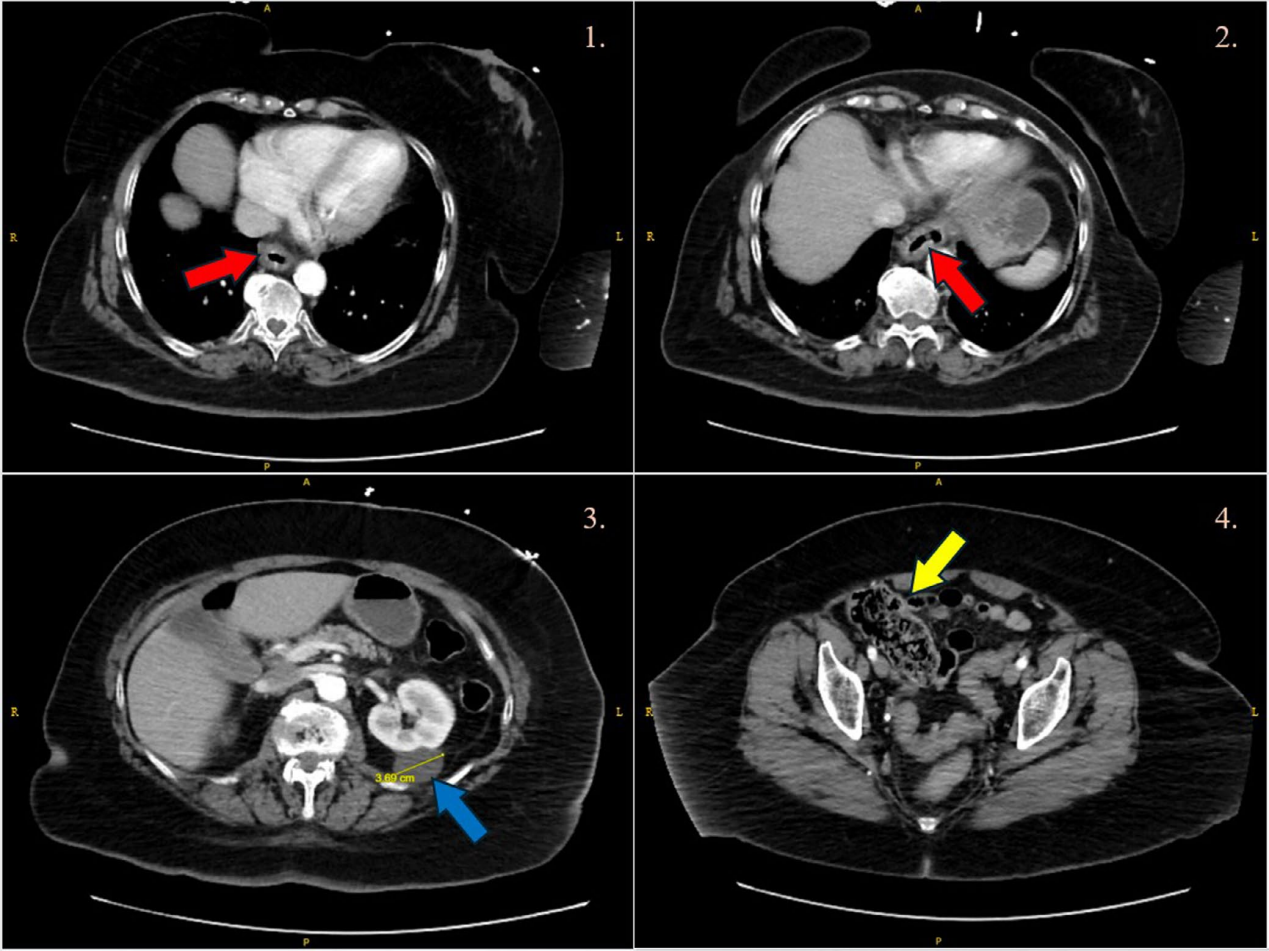
Contrast‐enhanced computed tomography of the abdomen and pelvis (axial sections) demonstrating wall thickening of the distal esophagus consistent with esophagitis (red arrows in panels 1 and 2), an incidental simple left renal cyst measuring 3.7 cm (blue arrow in panel 3), and uncomplicated sigmoid diverticulosis (yellow arrow in panel 4). No obstruction or intra‐abdominal infection was identified.

Contrast‐enhanced computed tomography of the abdomen and pelvis revealed wall thickening in the distal esophagus consistent with esophagitis, uncomplicated colonic diverticulosis, and an incidental 3.7 cm simple left renal cyst (Figure 2). There was no evidence of bowel obstruction, appendicitis, or intra‐abdominal infection.

The close temporal relationship between reinitiating semaglutide at 2.4 mg after a two‐month drug holiday and the appearance of symptoms strongly suggested drug‐induced gastrointestinal intolerance. The patient's condition was further complicated by hypertensive urgency with systolic blood pressure exceeding 210 mmHg, which responded promptly to labetalol and resumption of routine antihypertensives. Laboratory tests showed significant neutrophilic leukocytosis, initially raising concerns about infection; however, the patient remained afebrile with low procalcitonin and normal lactate levels. The leukocytosis resolved with supportive care, indicating a stress response rather than an infectious etiology. Hypokalemia from gastrointestinal losses was corrected with potassium supplementation.

A comprehensive workup systematically excluded gastroenteritis, bowel obstruction, pancreatitis, biliary disease, urinary tract infection, diabetic ketoacidosis, hyperosmolar hyperglycemic state, diverticulitis, and intracranial pathology. The final diagnosis was severe semaglutide intolerance following reinitiation at a high dose after a prolonged drug holiday, complicated by secondary esophagitis, stress leukocytosis, hypokalemia, and hypertensive urgency.

Management focused on supportive measures including discontinuation of semaglutide with thorough patient counseling about risks of re‐exposure, intravenous fluids and potassium repletion, cautious use of antiemetics considering QTc prolongation, pantoprazole for esophagitis, parenteral labetalol and resumption of home antihypertensives, and analgesia limited to acetaminophen to avoid worsening nausea. Empiric antibiotics were discontinued after infection was excluded.

The patient's symptoms progressively improved with supportive care and discontinuation of semaglutide. She was discharged with home health services and required 8 weeks of physical therapy due to significant deconditioning from the acute illness. She declined any rechallenge with semaglutide and was counseled about alternative weight management strategies.

## 3 Discussion

GLP‐1 RAs delay gastric emptying via vagal and enteric mechanisms, reducing antral motility and increasing pyloric tone [1, 2]. High doses or abrupt reinitiation after a drug holiday can cause profound gastric stasis, nausea, vomiting, and systemic complications such as esophagitis, electrolyte imbalance, and hypertensive urgency [3].

Our case highlights an unusual adverse effect pattern: acute onset of severe, multi‐system intolerance within hours of restarting semaglutide at 2.4 mg after a two‐month drug holiday, with symptoms resolving after medication discontinuation. This represents a rare presentation that differs from the more commonly described gradual development of gastrointestinal side effects during initial dose titration.

In our patient, hypokalemia and systemic stress responses likely prolonged the QTc interval observed on electrocardiography. Additionally, persistent vomiting contributed to the development of esophagitis, which was confirmed by CT imaging. The constellation of findings initially mimicked infectious or acute intra‐abdominal pathology, complicating the diagnostic approach and highlighting the importance of maintaining a high index of suspicion for medication‐related adverse effects in the appropriate clinical context.

Kashima and colleagues reported severe esophageal ulceration after several months of semaglutide‐induced vomiting, which resolved with withdrawal and proton pump inhibitors [4]. In contrast, our case demonstrates an acute presentation with rapid onset following reinitiation after a drug holiday, accompanied by systemic instability including hypertensive urgency and metabolic derangements. This underscores the broad spectrum of GLP‐1 RA‐related gastrointestinal toxicity and the particular risks associated with reinitiation at high doses without re‐titration.

Regulatory guidelines recommend gradual titration, close follow‐up during initiation, and individualized reassessment after adverse events [5, 6, 7]. However, specific guidance regarding reinitiation protocols after prolonged treatment interruptions remains limited. Our case suggests that a drug holiday may not eliminate intolerance risk and that re‐titration from lower doses may be prudent when restarting therapy, particularly in elderly patients with multiple comorbidities and polypharmacy.

For general internists and subspecialty physicians managing GLP‐1 RA therapy, this case emphasizes the importance of shared decision‐making, careful patient education about proper reinitiation protocols after treatment interruptions, and early discontinuation when severe intolerance occurs. Elderly patients with cardiovascular comorbidities may be at particular risk for systemic complications from severe gastrointestinal adverse effects.

This case emphasizes an important but rarely reported adverse effect pattern: severe, multi‐system complications following high‐dose semaglutide reinitiation after a prolonged drug holiday in an elderly patient. Key clinical implications for general internists and subspecialty physicians include considering re‐titration rather than resuming previously tolerated doses after treatment interruptions, recognizing that severe intolerance can occur within hours of administration, understanding that persistent gastrointestinal symptoms can precipitate acute metabolic derangements particularly in elderly patients with comorbidities, and ensuring prompt discontinuation when severe intolerance is identified. Clinicians should maintain a high index of suspicion for severe GLP‐1 RA intolerance in patients presenting with acute gastrointestinal symptoms following reinitiation, even if the medication was previously tolerated.

## Author Contributions

**Jay Mehta:** conceptualization, data curation, visualization, writing – original draft, writing – review and editing. **Brent Tai:** conceptualization, data curation, investigation. **Johnathan Frunzi:** supervision, writing – review and editing.

## Funding

The authors have nothing to report.

## Consent

Written informed consent was obtained from the patient for publication of this case report and accompanying images. Every effort has been made to protect the patient's identity, and all identifying information has been removed.

## Data Availability

Data sharing is not applicable to this article as no new data were created or analyzed in this study. This is a case report describing a single patient encounter, and all relevant clinical data are included within the manuscript and its tables and figures.

## References

[1] R. J. Jalleh , C. K. Rayner , T. Hausken , K. L. Jones , M. Camilleri , and M. Horowitz , “Gastrointestinal Effects of GLP‐1 Receptor Agonists: Mechanisms, Management, and Future Directions,” Lancet Gastroenterology & Hepatology 9, no. 10 (2024): 957–964. 39096914 10.1016/S2468-1253(24)00188-2

[2] Y. Nakatani , M. Maeda , M. Matsumura , et al., “Effect of GLP‐1 Receptor Agonist on Gastrointestinal Tract Motility and Residue Rates as Evaluated by Capsule Endoscopy,” Diabetes & Metabolism 43, no. 5 (2017): 430–437. 28648835 10.1016/j.diabet.2017.05.009

[3] R. J. Jalleh , M. P. Plummer , C. S. Marathe , et al., “Clinical Consequences of Delayed Gastric Emptying With GLP‐1 Receptor Agonists and Tirzepatide,” Journal of Clinical Endocrinology and Metabolism 110, no. 1 (2025): 1–5. 10.1210/clinem/dgae719 PMC11651700 39418085

[4] S. Kashima , K. Moriichi , and M. Fujiya , “Severe Reflux Oesophagitis Caused by Repeated Vomiting due to Glucagon‐Like‐Peptide‐1 Receptor Agonist,” BMJ Case Reports 18 (2025): e266368. 10.1136/bcr-2025-266368 PMC12322966 40759494

[5] A. Spitery , M. J. Elder , N. Farhat , I. Mohammad , and A. Lobkovich , “Legal, Safety, and Practical Considerations of Compounded Injectable Semaglutide,” Journal of the American College of Clinical Pharmacy 7, no. 9 (2024): 941–946.

[6] M. M. Smits and D. H. Van Raalte , “Safety of Semaglutide,” Frontiers in endocrinology 12 (2021): 645563. 34305810 10.3389/fendo.2021.645563 PMC8294388

[7] J. Morales , J. H. Shubrook , and N. Shkolnik , “Practical Guidance for Use of Oral Semaglutide in Primary Care: A Narrative Review,” Postgraduate Medicine 132, no. 8 (2020): 687–696. 32643514 10.1080/00325481.2020.1788340

